# The Many Facets of Hypoxia in Plants

**DOI:** 10.3390/plants9060745

**Published:** 2020-06-12

**Authors:** Elena Loreti, Pierdomenico Perata

**Affiliations:** 1 Institute of Agricultural Biology and Biotechnology, CNR, National Research Council, Via Moruzzi, 56124 Pisa, Italy; 2 PlantLab, Institute of Life Sciences, Scuola Superiore Sant’Anna, Via Giudiccioni 10, 56010 San Giuliano Terme, 56124 Pisa, Italy

**Keywords:** anaerobiosis, anoxia, Arabidopsis, flooding, hypoxia, rice, submergence, waterlogging, development

## Abstract

Plants are aerobic organisms that require oxygen for their respiration. Hypoxia arises due to the insufficient availability of oxygen, and is sensed by plants, which adapt their growth and metabolism accordingly. Plant hypoxia can occur as a result of excessive rain and soil waterlogging, thus constraining plant growth. Increasing research on hypoxia has led to the discovery of the mechanisms that enable rice to be productive even when partly submerged. The identification of Ethylene Response Factors (ERFs) as the transcription factors that enable rice to survive submergence has paved the way to the discovery of oxygen sensing in plants. This, in turn has extended the study of hypoxia to plant development and plant–microbe interaction. In this review, we highlight the many facets of plant hypoxia, encompassing stress physiology, developmental biology and plant pathology.

## 1. Hypoxia and Its Sensing

Oxygen availability is a pre-requisite for life in several living organisms. In plants, when the oxygen supply is insufficient, most cellular functions are compromised, which can lead to death [1]. In order to keep the level of oxygen under control, cells sense the oxygen levels and react to insufficient oxygen (hypoxia) by adopting survival strategies ranging from gene regulation to morphological adaptive responses [2].

Hypoxia occurs when the oxygen level limits aerobic respiration (usually between 1% and 5%), while anoxia takes place when oxygen is absent in the environment [3]. It is also important to highlight the difference between acute and chronic hypoxia [4]. The term acute hypoxia is used when the drop in oxygen availability is transitory in plants, which can be due to adverse environmental conditions such as flooding events or when unusual, transient increases in oxygen consumption occur in a plant tissue. Acute hypoxia is perceived by the plants as stressful. On the other hand, chronic hypoxia is a constitutive and, usually, non-stressful condition where the oxygen level is maintained low only in a given group of cells and not in the entire plant. In other words, chronic hypoxia can be a physiological condition in specific plant tissues. 

The molecular mechanism of oxygen perception has been revealed both in animals and plants. In mammals, the Hypoxia Inducible Factor (HIF-1) is responsible for low-oxygen sensing, while this molecular activity is controlled by the Cys branch of the N-degron pathway in plants [5,6]. 

HIF-1 is formed by two subunits: HIF-1β is constitutively expressed, while HIF-1α is regulated by oxygen through post-translational modifications [7,8,9]. During normoxia, HIF-1α is degraded via the ubiquitin–proteasome pathway, whereas during hypoxia, it is stabilized and protected from proteolysis, allowing its migration into the nucleus [10]. Here, the complex HIF1α and HIF-1β is reconstituted [7], which induces the transcription of hypoxic genes. 

**Figure 1:**
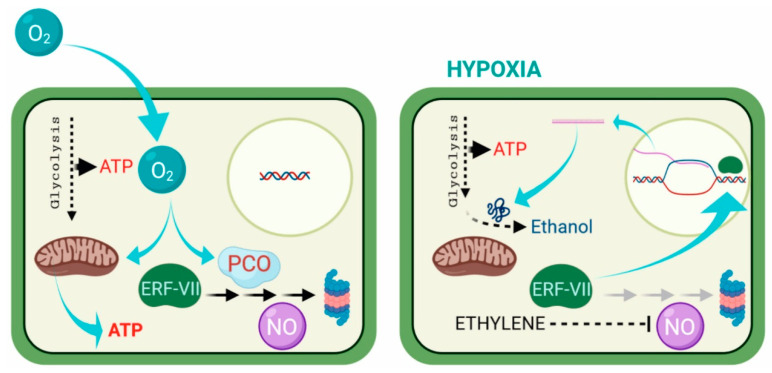
How plants sense oxygen. Under aerobic conditions (left), aerobic respiration in the mitochondria provides most of the energy (ATP) required for the cell metabolism. The ERF-VII transcription factor genes are constitutively expressed, but their stability is compromised by the activity of PCOs, which, in a process requiring oxygen, oxidize the N-terminal Cys residue, channeling the ERF-VII proteins to the proteasome, in a process also requiring nitric oxide (NO). Under hypoxia (right), the respiration in the mitochondria is drastically reduced, and ATP production can only occur because of enhanced glycolytic activity. The ERF-VII proteins are stabilized because of the absence of oxygen and also thanks to ethylene production, which dampens the presence of NO in the cell. The stable ERF-VII proteins migrate to the nucleus where they activate the transcription of Hypoxia-Responsive Genes (HRGs), including genes encoding proteins required for alcoholic fermentation. This figure was created using BioRender [17].

The oxygen-sensing mechanism in plants (Figure 1) was revealed several years later after the discovery of HIF-1 [5,11,12]. In plants, proteins belonging to group VII of the Ethylene Response Factors (ERF-VIIs) are destabilized by oxygen and become stable only under hypoxia, with a mechanism conceptually similar to the one that regulating HIF-1. ERF-VIIs are transcription factors characterized by a Cys residue at their N-terminal [13]. Under normoxia, the Cys residue is constitutively oxidized by a class of plant enzymes named Plant Cysteine Oxidases (PCOs), which target the ERF-VII proteins to degradation via the proteasome, following the Cys branch of the N-degron pathway [14,15]. Under hypoxia, on the other hand, ERF-VIIs are stable, given that the absence of oxygen prevents the oxidization of the Cys residue by PCOs. The stabilized ERF-VIIs can translocate to the nucleus where they activate the transcription of anaerobic genes by binding to the Hypoxia-Responsive Promotor Element (HRPE) present in the promoter of anaerobic genes [16]. 

Once oxygen deficiency has been perceived, plants change their metabolism, which is also achieved by a modulation in gene expression [18]. In Arabidopsis, 49 anaerobic core hypoxic genes are induced in all plant organs during a hypoxic event [18].

Although oxygen-sensing mechanisms in mammals and plants are different, an overlapping sensing machinery between plants and the animal kingdom was recently discovered [19]. Mammals possess a cysteamine dioxygenase enzyme, named ADO, which is similar to the PCOs of plants and operates similarly on proteins possessing a Cys residue at the N-terminus [19]. With their differences and similarities [12], oxygen-sensing mechanisms in animals and plants represent the first steps in the cascade of events following hypoxia. 

## 2. Adaptation to Hypoxia at the Cellular Level

Aerobic organisms have developed a variety of adaptive responses to hypoxia at the cellular, tissue and organism levels [20]. When the oxygen supply is adequate, the mitochondria produce enough ATP for survival, while under hypoxia alcoholic fermentation replaces mitochondrial respiration [21]. The aim of the fermentative metabolism is to allow ATP production through the glycolytic pathway by recycling NAD+ via the action of two key enzymes, pyruvate decarboxylase (PDC) and alcohol dehydrogenase (ADH) [21]. These two enzymes belong to the class of anaerobic polypeptides (ANPs), which are induced and produced under hypoxia [21]. These are produced through the action of ERF-VIIs, which activate the transcription of the Hypoxia-Responsive Genes (HRG) that encode the ANPs.

In addition to those involved in the fermentative pathway, ANPs also include proteins linked to aerenchyma formation, cytoplasmatic pH and carbohydrate metabolism [2,22]. Carbohydrate degradation through glycolysis coupled with the fermentative pathway leads to 2 moles of ATP instead of the 36 normally produced during aerobic respiration [21]. Although limited, ATP production through fermentation is important for hypoxia tolerance [1]. Moreover, mutants defective in alcoholic fermentation are intolerant to hypoxia, demonstrating that this pathway contributes significantly to hypoxia tolerance [23]. In order to be efficient at ATP production, glycolysis coupled to fermentation under low oxygen availability requires an adequate glucose supply [1,24]. In this context, the role of starch as a source of sugars to be used during prolonged hypoxia has been demonstrated, both in cereals and in Arabidopsis [1,24]. Of the cereals, only rice is able to germinate under anoxia due to its ability to exploit the starchy reserves present in the caryopses, a consequence of the successful induction of α-amylases even in the absence of oxygen [25,26]. 

These key enzymes, which are required for starch degradation, are not produced in other cereals, which results in their inability to germinate under hypoxia [27,28]. Although Arabidopsis tolerance to hypoxia depends on distinct mechanisms that enable rice seeds to germinate, starch is also an essential component of the anaerobic response in this species. Arabidopsis adult plants require starch for their tolerance to submergence and, interestingly, a relation between sugar starvation and the plant’s ability to induce HRG transcription has been observed [29]. Low-oxygen conditions weaken the anaerobic response at the transcriptional level, indicating the existence of a homeostatic mechanism linking oxygen sensing with sugar sensing, which controls the intensity of the induction of HRGs, so that this matches the available carbon resources [29]. 

## 3. Environmental Hypoxia

Hypoxia can be caused by specific environmental conditions, which we refer to as “environmental hypoxia”. In humans, a lack of oxygen can be due to environmental hypoxia at high altitudes, where the partial oxygen pressure (pO_2_) is decreased in proportion to the lower ambient pressure [30]. Besides environmental hypoxia, pathological conditions such as chronic obstructive pulmonary disease [31], obstructive sleep apnea [32] and anemia [33] can also lead to hypoxia. Similarly, during intense exercise, hypoxia can also be generated by the intense respiratory metabolism [34].

**Figure 2:**
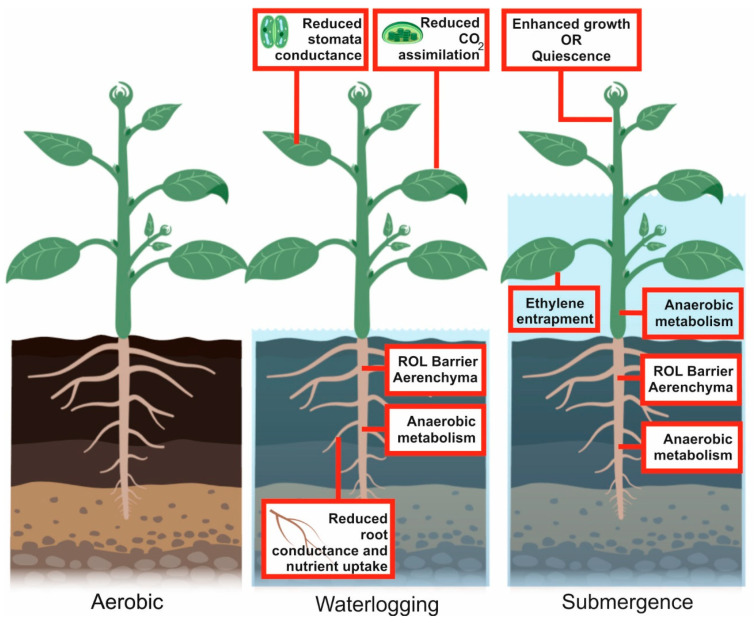
Environmental hypoxia is generated by soil waterlogging or by plant submergence. An excess of water in the soil reduces aerobic respiration, which is replaced by anaerobic metabolism. The uptake of nutrients is reduced. A radial oxygen loss (ROL) barrier and aerenchyma eventually develops in the root system to provide oxygen to the submerged roots. The aerial part of the plant, although not submerged, is also affected by waterlogging, with reduced stomata conductance and CO_2_ assimilation. Complete or partial plant submergence may trigger quiescence or escape strategies, characterized by reduced or enhanced elongation, respectively. Ethylene entrapment in the submerged plant plays an important role in defining the overall plant response to submergence. Aerenchyma develops and the metabolism is mostly anaerobic, with the exception of tissues and organs that receive sufficient oxygen through the aerenchyma. This figure was created using BioRender [17].

In plants, hypoxia often originates during flooding, leading to waterlogging or submergence [1] (Figure 2). Waterlogging is the saturation of soil with water and occurs when roots cannot respire due to a water excess, whereas the term submergence means that, in addition to the roots, the aerial part of the plant is under water [3]. In both cases, the final effect is a lack of oxygen. 

How plants survive flooding has been studied for decades and, besides the molecular responses described above, survival also entails morpho-physiological modifications such as aerenchyma development, formation of barriers to radial oxygen loss, production of adventitious roots, elongation of stems, and leaf petioles (Table 1). 

Overall, plants respond to environmental hypoxia through different strategies, aimed at prolonging their life even under these unfavorable conditions [2]. Some plant species respond to submergence by attempting to escape from the low-oxygen environment by elongating their stems, petioles or leaves so that at least part of the plant is above water. This facilitates the transport of oxygen to the submerged organs, by means of the aerenchyma, whose development is usually enhanced by submergence [2]. The production of ethylene, entrapped by the water surrounding the submerged plant, plays an important role and regulates several aspects of the enhanced growth, enabling the plant to escape submergence [52]. Plant species that adopt the escape strategy include wild species, such as *Rumex palustris*, and crop plants, such as some rice varieties [2]. 

The escape strategy is energetically costly. It is only advantageous if the rate of elongation is fast enough to keep part of the plant above the water surface. If, instead, growth is enhanced but is insufficient for the plant to reach the surface of the submerging water, then this strategy is detrimental to survival, since the plant will use most of its reserve carbohydrates to fuel growth and may be starved of carbon when the water recedes and normal plant physiology should be re-established. Some plant species are very well adapted to prevent the starvation syndrome arising from submergence, and, instead of enhancing growth, they adopt a quiescence strategy based on very limited growth when the plant is submerged. 

Rice is a wetland species that has evolved in very distinct environments, very often characterized by phases of growth suffering from flooding events. Some rice species (known as deep-water rice or floating rice), have adapted well to environments where submergence is prolonged and deep [46,53]. Deep-water rice survives due to a set of genes, named *Snorkel*, which are ethylene-responsive and trigger a very fast elongation response [53]. Other flooding-resistant rice varieties, also known as “Scuba-rice”, can tolerate short-term complete submergence by adopting a quiescence strategy made possible by the *Sub1A* gene. *Sub1A* is ethylene-responsive but, unlike the *Snorkel* genes, triggers reduced elongation, thus allowing the plant to preserve its carbohydrate reserves, which are required to ensure vigorous re-growth when the water recedes [48,49]. 

The regulation of gene expression is thus important during environmental hypoxia because it controls the induction of genes that are required for tolerance. The cellular events previously described occur during environmental hypoxia in all those tissues which, because of submergence, do not receive an adequate oxygen supply, but not in tissues that become aerated because of oxygen transport via the aerenchyma to the submerged organs.

## 4. Developmental Hypoxia

In humans, hypoxia can arise as a consequence of impaired blood flow. This can significantly damage organ structure and function, resulting, for example, in a stroke (cerebral ischemia) or heart infarction (myocardial ischemia). Hypoxia also regulates tumor growth and metastasis [54].

Although, in plants, hypoxia traditionally refers to the stress due to flooding events, it can also occur in tissues and organs under normal oxygen availability. In this case, hypoxia represents a physiological status that is experienced by the plant throughout its life or during a specific stage of development. Oxygen diffuses from the air (21% [*v*/*v*] oxygen) into the plant body across apertures in the epidermis and through intracellular air spaces within a tissue [36]; however, unlike in animals, it is still unknown if plants have an efficient mechanism that distributes oxygen to cells. As a consequence, plants have to deal with oxygen gradients in tissues and organs [4]. 

The anatomy of specific tissues or organs such as in seeds, fruits and roots may limit oxygen diffusion, leading to hypoxia [55,56,57]. In bulky organs, the oxygen concentration can be very low in the inner part of the organ [58], thus limiting oxygen for respiration. For example, oxygen concentration in the center of growing potato tubers can decrease to around 5%, thus making this tissue hypoxic [59]. 

With the notable exception of bulky fruits and organs, most plant organs possess a relatively high surface-to-volume ratio that should allow oxygen diffusion, thus preventing the establishment of hypoxia [55]. Even tissues in which oxygen diffusion is not problematic may become hypoxic when there is high metabolic activity, especially if there are no intercellular air spaces. High local rates of oxygen consumption may take place in tissues such as the phloem, which has been shown to be hypoxic [55]. Furthermore, as discussed by van Dongen et al. [55], oxygen access might be restricted because phloem is a dense tissue with few intercellular spaces. Measurements of oxygen profiles across stems of *Ricinus communis* plants showed that oxygen levels can be as low as approximately 7% (*v*/*v*) in the vascular regions, even when plants are grown under fully aerobic conditions [55]. 

Interestingly, alcohol dehydrogenase activities, as well as the presence of ethanol, have been observed in the vascular cambium of trees [60], supporting the existence of hypoxic tissues near the phloem. Physiological hypoxia usually occurs in heterotrophic tissues, given that autotrophic tissue produces photosynthetic oxygen and can therefore more easily prevent the occurrence of hypoxia. During germination, reduced oxygen availability in soil plays a positive role in the survival and establishment of seedlings following darkness, through the enhanced stability of ERF-VIIs, which enhance dark-activated development and repress light-activated development [61].

Environment-independent hypoxia can be limited to a small cluster of cells or to entire organs. In animals, the formation of a hypoxic microenvironment occurs when the cells need to be kept in an undifferentiated state, such as in stem cells [62,63]

Interest in hypoxic microenvironments is also growing in plant sciences. Pollen can be hypoxic [64], and, in fact, given that hypoxia arises naturally within growing anther tissue, it acts as a positional cue to establish germ cell fate [65]. 

**Figure 3:**
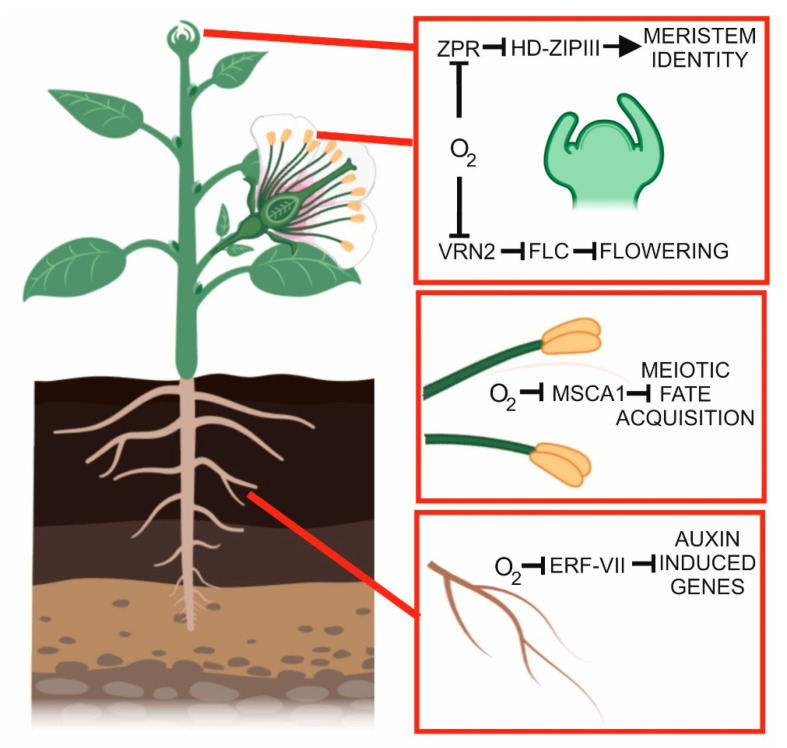
Hypoxic niches affecting developmental processes. In the roots, hypoxia in the lateral root primordia affects auxin-regulated genes through the action of ERF-VII proteins. In the anthers, hypoxia represents a developmental signal influencing meiotic fate acquisition (MSCA: male sterile converted anther 1). In the shoot apical meristem, a hypoxic niche controls the stability of ZPR2 and VRN2, affecting meristem identity and flowering, respectively. This figure was created using BioRender [17].

The concept of hypoxia as a developmental, positional cue was recently strengthened (Figure 3), and hypoxic niches were shown to be present in shoot apical meristems (SAM; [66]). Using a micro-scale oxygen electrode, Weits et al. [66] measured the oxygen concentration inside the shoot apical meristems of Arabidopsis (which are very small) and that of *Solanum lycopersicum* (which are larger). In both cases, the oxygen concentration inside the meristem was below 5%, highlighting that hypoxia niches are a conserved occurrence in plants. Interestingly, Weits et al. [66] demonstrated that low oxygen in the SAM is not only a non-stressful condition, but is actually required for the production of new leaves [66]. This is because, in SAM-related proteins, a novel substrate of the Cys branch of the N-degron pathway, namely, LITTLE ZIPPER 2 (ZPR2), due to its hypoxia-dependent stabilization, drives leaf organogenesis in a hypoxia-dependent manner by interacting with its target class III homeodomain leucine zipper (HD-ZIP III) proteins [66]. The hypoxic niche at the shoot meristem not only promotes the stability of ZPR2, but also of VERNALIZATION 2 (VRN2; [67]), which requires low oxygen to be stable and thus regulates reproductive and vegetative development by acting on its target FLOWERING LOCUS C (FLC) [67]. 

Hypoxia in the SAM triggers the stabilization of the ERF-VII transcription factors, which drive the expression of the core hypoxic genes, including those involved in the fermentative pathway such as ALCOHOL DEHYDROGENASE (ADH) and PYRUVATE DECARBOXYLASE (PDC). These genes are induced not only in SAM but also in the Lateral Root Primordia (LRP) of Arabidopsis, suggesting that also LRP are hypoxic [68]. Low oxygen influences root architecture, such as the formation of adventitious roots [69], the promotion of root bending and the inhibition of primary roots [70]. Physiological hypoxia occurs during LR development irrespectively of the oxygen concentration in the environment [68]. The consequence of the formation of hypoxic niches during lateral root primordia development is the stabilization of ERF-VII transcription factors, which induce the set of hypoxic genes and, at the same time, the downregulation of crucial auxin-induced genes that promote lateral root production [68].

Roots are, in fact, the organs most affected by limited oxygen supply when the plants are waterlogged or submerged. Hypoxia causes the primary roots to grow sideways, possibly to escape soil patches with reduced oxygen availability via ERF-VII activity and mediated by auxin signaling in the root tips [70].

It is still unclear how hypoxic conditions are established in the SAM and in root primordia. While the slow oxygen diffusion rate through plant tissues explains why bulky plant organs are hypoxic, it is less obvious why oxygen levels drop to very low levels in plant meristems. One hypothesis is the possibly very active respiratory metabolism in the SAM, which makes the rate of oxygen diffusion insufficient to cope with oxygen consumption in the cells. Alternatively, the presence, yet to be demonstrated, of barriers to oxygen diffusion may keep the SAM hypoxic.

## 5. Hypoxia in Plant–Microbe Interactions

In legumes, the capacity to establish a symbiotic relationship with endosymbiotic dinitrogen-fixing rhizobia requires the establishment of hypoxic conditions, given that the activity of the nitrogenase enzyme, which can fix N_2_, is inactivated by free O_2_. Nodules are able to maintain an internal low-O_2_ environment, including a nodule O_2_ diffusion barrier, and by expressing O_2_-carrying symbiotic plant hemoglobins (reviewed by Pucciariello et al. [71]). 

In other plant species, hypoxia-responsive genes have been reported to be induced in roots infected by *Plasmodiophora brassicae* [72] or in stems infected by *Agrobacterium tumefaciens* [73], suggesting that hypoxic conditions are generated at the sites of infection by these bacteria. Slower O_2_ diffusion rates in tumorigenic tissues, such as clubroot, and in crown galls, may be responsible for the establishment of hypoxia in these conditions [74]. Alternatively, intracellular competition for O_2_ between the host and the pathogen can reduce the oxygen available to the plant and lead to hypoxia [72]. In the case of crown galls, the increased respiration rate in the plant cells, which is required to fuel rapid cell proliferation rates, may be the cause of the hypoxic conditions [73].

The evidence of a possible role of ERF-VII proteins, which require hypoxia to be stable, in pathogen resistance has raised the question as to whether hypoxia is established during pathogen infection [72,75,76,77]. Low-oxygen conditions may be established during pathogen infection, as described above, or by modulation of nitric oxide (NO) levels, as NO is another ERF-VII de-stabilizing molecule [72,78]. Given that ethylene is often involved in plant–pathogen interaction, the NO-scavenging activity of the ethylene-induced PHYTOGLOBIN1 (PGB1) may also play a role which has not yet been studied [79]. In fact, hypoxia is present in leaves that are infected with *Botrytis cinerea* [80]. The hypoxic conditions are established locally, at the exact sites of *B. cinerea* infection, where an oxygen concentration of less than 1% has been measured. Given that leaves are usually fully aerobic tissues, also due to their ability to produce oxygen through photosynthesis, the establishment of hypoxia suggests that competition of oxygen at the sites of infection may be very strong. Although increased oxygen consumption was actually measured at the sites of *B. cinerea* infection, it is still not clear whether it is fungal or plant respiration that is enhanced to a level that leads to the rapid depletion of oxygen availability [80]. 

In addition to *B. cinerea*, infection by *Alternaria brassicicola*, but not *Pseudomonas syringae*, also leads to the establishment of hypoxia in leaves. Besides playing a role in enhanced respiration in the infected tissue, hypoxia arising from infection by necrotrophic fungi can also be the consequence of tissue waterlogging in the necrotic area [80]. An important question is thus whether hypoxia contributes to plant tolerance to *B. cinerea* infection or is actually an advantage for the fungus. Given that ERF-VII proteins can contribute to *B. cinerea* resistance in Arabidopsis [75] and that these proteins require hypoxia to be stable and active, it is tempting to speculate that the low-oxygen environment that is established at the site of infection is advantageous for the plant. The stability of RAP2.3 (an ERF-VII) makes it possible to interact with OCTADECANOID-RESPONSIVE ARABIDOPSIS 59 (ORA59) [76], thus enhancing tolerance to the fungus. 

**Figure 4:**
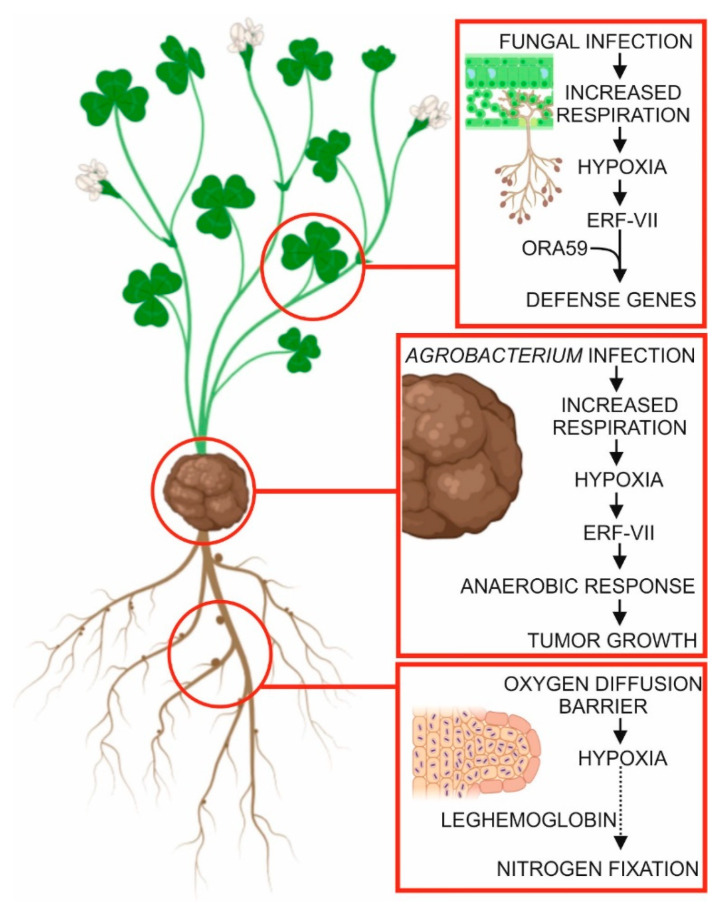
Local hypoxia establishment during plant–microbe interactions. Leaf infection by necrotrophic fungi results in enhanced respiration, which eventually leads to hypoxia. Under these conditions, ERF-VII proteins are stable and, through interaction with ORA59, they influence the efficacy of a plant’s response to the pathogen. Similarly, agrobacterium infection enhances respiration in the infected tissue, which leads to hypoxia. Stable ERF-VII activates the anaerobic response that favors tumor growth. In legumes, rhizobia requires hypoxic conditions, given that the activity of nitrogenase enzyme, which can fix N_2_, is inactivated by oxygen. Nodules maintain an inner low-O_2_ environment thanks to the presence of a nodule O_2_ diffusion barrier and by expressing O_2_-carrying symbiotic plant hemoglobins (leghemoglobin). This figure was created using BioRender [17].

The plant’s response to fungal infection includes the production of elicitor molecules that are aimed at boosting the plant’s response to the pathogen. Oligogalacturonides (OGs) are important elicitors in the response to *B. cinerea* infection. Interestingly, the fate of OGs as elicitors might depend on the establishment of local hypoxia at the site of *B. cinerea* infection [80]. OGs can be oxidized by Arabidopsis berberine bridge enzyme-like proteins (OXOGs) [81]. Oxidized OGs are less able to trigger the immune responses in the plant and are less hydrolysable by fungal polygalacturonases [81]. Hypoxia could therefore be beneficial for the fungus because it prevents the oxidation of OGs that would otherwise be oxidized and are no longer metabolically useful for the fungus. On the other hand, the plant benefits from hypoxia, which prevents oxidation of OGs making them less effective as elicitors. The overexpression of OXOGs results in enhanced resistance to *B. cinerea* [81], suggesting that the fungus takes advantage of OGs as a metabolic source of carbon. Establishing whether it is the fungus or the plant that benefits from hypoxia at the site of infection thus remains an open question (Figure 4).

## 6. Concluding Remarks

The hypoxia field of study in animal physiology is quite broad, ranging from sensing at the cellular level to its role in pathogenesis. In plants, for decades, research on hypoxia has been mostly restricted to how plants respond to adverse environmental conditions such as excessive rain, resulting in soil waterlogging and plant submergence. Although hypoxia is of great relevance for agriculture, also due to the challenges of climate change, plant hypoxia has been erroneously considered a minor abiotic stress compared to, for example, drought.

Understanding the mechanisms enabling rice plants to survive submergence, involving ERF-VII transcription factors, has paved the way to the discovery of oxygen sensing in plants. Several papers in this research area have recently extended hypoxia research to other fields, including developmental biology and plant pathology. 

During plant–microbe interactions, hypoxia is required for generating an environment that is compatible with oxygen-sensitive enzymatic activities, such as during nitrogen fixation in leguminous plants. On the other hand, hypoxia at the site of infection of necrotrophic pathogens may be required to activate oxygen-sensitive plant resistance pathways, or it may alter the production and stability of elicitors in a hypoxia-dependent manner.

The identification of new targets of the oxygen-dependent N-degron pathway unequivocally demonstrates that the absence of oxygen has profound effects on the physiology and developmental biology of the plant, well beyond the metabolic adaptive response.

The existence of hypoxic niches in an otherwise aerobic plant body raises a number of issues. Firstly, we need to know how hypoxia is achieved in hypoxic niches. Secondly, it is of importance to assess how many enzymatic activities are affected in hypoxic niches, including those involved in the synthesis of plant hormones, with consequences for the hormone concentration gradients. Thirdly, we need to assess the energy status of hypoxic niches and the impact of energy sensing on developmental processes. How is hypoxia achieved in hypoxic niches? The limited diffusion of oxygen though tissues and intense respiratory activity are good candidates for explaining developmental hypoxia; however, experimental evidence is missing. The existence of a gene family of PCOs with different affinities for oxygen and, possibly, distinct expression patterns across different cell types is another fascinating aspect of oxygen sensing in plants, which may provide evidence of a much more complex regulatory network for hypoxia-modulated processes in plants. Besides PCOs, limited oxygen availability will inevitably influence the activity of several other enzymes, including those involved in the synthesis of plant hormones, but also as-yet-unidentified oxygen-requiring enzymes whose activity will be restricted in hypoxic niches. The identification and evaluation of these enzymes is of importance, as they may represent ancillary oxygen-sensing mechanisms in specific plant tissues. Finally, how is metabolism adjusted so that energy production is not compromised during chronic hypoxia? Is fermentation sufficient? All these questions await experimental evidence. 

## Figures and Tables

**Table 1:** Traits affected by waterlogging and submergence in plants.

Trait	Function	Plant Species	References
Aerenchyma	Improvement in internal gas diffusion	*Zea mays*; *Oryza sativa*; *Pisum sativum*; *Triticum aestivum*; *Arabidopsis thaliana*	[2,35,36,37,38,39,40]
Hypertrophic lenticels	Facilitating O_2_ diffusion; venting ethylene and CO_2_	Woody plant species	[41]
Radial oxygen loss barrier	Barrier impermeable to radial O_2_ loss	*Oryza sativa; Phragmites australis; Phalaris aquatica*	[37,41,42]
Increased specific leaf area (indicating a large surface area relative to mass)	CO_2_ enters the mesophyll cells via diffusion through the epidermis and not via stomata	*Rumex palustris* and other amphibious species	[2]
Petiole elongation	Reaching water surface	*Rumex palustris*	[2,43]
Reorientation of petioles in upright position	Reaching water surface	*Rumex palustris*	[2,43]
Coleoptile elongation	Reaching water surface	*Oryza sativa*	[26,44,45]
Fast stem elongation	Reaching water surface	*Oryza sativa* (deep water rice)	[46,47]
Inhibition of stem elongation	Reducing growth-associated costs (quiescence strategy)	*Oryza sativa*	[48,49]
Root architecture	Minimize the distance between the aerial surface and the flooded root tips	*Oryza sativa*; *Zea mais*; *Triticum aestivum*	[1,38]
Adventitious roots production	Replace primary root systems; roots at surface of water; enhance supply of water and minerals	*Zea mais*; *Solanum lycopersicon*; *Solanum dulcamara*	[50,51]
